# Osteopathy in the French-speaking part of Switzerland: Practitioners’ profile and scope of back pain management

**DOI:** 10.1371/journal.pone.0232607

**Published:** 2020-05-01

**Authors:** Anne-Sylvie Bill, Julie Dubois, Jérôme Pasquier, Bernard Burnand, Pierre-Yves Rodondi

**Affiliations:** 1 Center for Primary Care and Public Health (Unisanté), University of Lausanne, Lausanne, Switzerland; 2 Institute of Family Medicine, University of Fribourg, Fribourg, Switzerland; Charles Sturt University, AUSTRALIA

## Abstract

**Background:**

Osteopathy is commonly used for spinal pain, but knowledge about back pain management by osteopaths is scarce.

**Objective:**

The aim of this study was to survey osteopaths across the French-speaking part of Switzerland about the scope of their practice and their management of patients with back pain.

**Design:**

This cross-sectional observational study was based on an online survey conducted from March to June 2017. **Setting and participants**: All registered osteopaths of the French-speaking part of Switzerland were asked to complete the survey. **Outcome measures**: In addition to descriptive statistics (practice characteristics, patients’ profiles, scope of treatment modalities, health promotion, research, and osteopathic practice), we explored variables associated with osteopaths’ practice, such as age and gender.

**Results:**

A total of 241 osteopaths completed the questionnaire (response rate: 28.8%). Almost two thirds of osteopaths were female. Ages ranged from 25 to 72 years with an overall mean of 42.0 (SD 10.7) years. Male osteopaths reported more weekly working hours than female osteopaths did (38.2 [SD 11.0] vs 31.6 [SD 8.9], respectively, p<0.001). Almost a third (27.8%,) of osteopaths could arrange an appointment for acute conditions on the same day and 62.0% within a week. Acute or subacute spinal conditions, mainly low back and neck pain, were the most frequent conditions seen by our respondents. For 94.4% of osteopaths, one to three consultations were required for the management of such conditions.

**Conclusion:**

Osteopaths play a role in the management of spinal conditions, especially for acute problems. These findings, combined with short waiting times for consultations for acute conditions, as well as prompt management capabilities for acute low back and acute neck pain, support the view that the osteopathic profession constitutes an added value to primary care.

## Introduction

Osteopathy has gained popularity over the past decades and is recommended in several national guidelines as a management option for low back pain [1–4]. Access to osteopathy within the healthcare system varies from one country to another. In the United States, osteopathic physicians have a full medical license and are therefore fully integrated in the healthcare system [5]. In Europe, osteopathy ranges from allied to complementary medicine (CM) and its regulation varies between countries. The majority of recommendations for osteopathic practice originate from countries such as the United States [6, 7], Australia [8–10]), and Canada [11]. In Europe, the profiles of osteopathic practices and patients have been described in the United Kingdom [12, 13], Spain [14], the Benelux (Belgium, Netherlands, and Luxembourg) [15], and recently Switzerland [16] and Italy [17]. In these studies, most patients attending osteopathic consultations presented with low back pain, and the most common treatment techniques used by osteopaths were soft tissue techniques.

The yearly use of osteopathy in the population aged 15 and older is slightly rising in Switzerland, as it was reported to be 5.4% in 2007 [18] and 6.2% in 2012 [19]. Moreover, osteopathy was the third most used CM in the general population in 2012 [19]. In 2017, there were 1222 registered osteopaths in Switzerland [20]. By comparison, there were approximately 359 chiropractors and 1311 medical doctors with certified training in manual medicine [21].

In Switzerland, the osteopathic profession is in the process of transition. Since 2006, in order to set standards in their professional competences, practitioners with a diploma in osteopathy (D.O) and a two-year internship have been invited to take a two-stage examination at the Swiss Conference of Cantonal Health Directors (GDK-CDS) [22]. Since then, the majority of Swiss cantons have delivered new licenses to practice osteopathy only to osteopaths with a GDK-CDS diploma. In the autumn of 2014, a curriculum in osteopathy was developed at the master’s level [23]. In 2016, osteopathy was recognized as a primary healthcare profession in Switzerland [24]. In January 2020, the cantonal regulations were replaced by a national regulation that will allow only those with a GDK-CDS diploma or a master’s degree to set up practice [24].

Despite its recognition as a primary healthcare profession in Switzerland, osteopathy is not yet covered by mandatory basic health insurance but is part of private supplemental insurance plans. Health insurance companies provide a wide range of supplemental insurance options with no guarantee of admission and only partial reimbursement. To date, private supplemental insurance plans have covered osteopaths with a GDK-CDS diploma, a master’s degree in osteopathy, or a D.O under the same conditions.

In Switzerland, a detailed practice review was conducted in 2017 [16], which offered an overview of Swiss osteopathic practice throughout Switzerland. Although both studies explore some common features in terms of socio-demographic and practice characteristics, ours further explores variables associated with osteopaths’ practice, such as age and gender, and provides new information, in particular about osteopaths’ management of back pain. As our study is limited to the French-speaking part of Switzerland, a comparison with their results will either confirm their findings or underline differences that might be linked to different work cultures in the areas considered. The purpose of this study was thus to survey osteopaths across the French-speaking part of Switzerland and to define their profile, practice activities, and main reason for consultation, as well as to describe their management of patients with back pain. As a secondary objective, we explored variables associated with osteopaths’ practice, such as age, gender, years in practice, and having a GDK-CDS diploma.

## Methods

### Study design

This cross-sectional observational study was based on an online, anonymous, practice-based survey conducted between March and June 2017. The survey was sent to all osteopaths recorded in at least one of these registries: EMR (empirical medicine register) and ASCA (Fondation Suisse pour les médecines complémentaires). The detailed study design is explained elsewhere [25]; however, in the present study, the inclusion criteria are different because osteopaths with a medical degree were included. The Cantonal Commission for the Ethics of Human Research (CER-VD) approved the study (Reference Req-2016-00535) and waived the need for consent, given the scope and nature of the study and the anonymous data collection and analysis.

### Settings and participants

All registered osteopaths practicing in the western part of Switzerland and having French as their language of correspondence were eligible for the study (n = 838). GDK-CDS diploma holders, non- GDK-CDS diploma holders (including assistant osteopaths and osteopaths with a D.O), and physicians with an osteopathic diploma were included.

### Variables and data sources

We developed an online questionnaire by using LimeSurvey software on the basis of the literature and existing surveys [12, 13, 26, 27]. It was divided into two parts. The first of these was part of a larger study explained elsewhere [25], in which we collected information on practice characteristics, work environment and workload, patients’ main concerns, transmission of information, and treatment coverage. The second part was dedicated to osteopathy, including the scope of treatment modalities, and, specifically, low back pain management. The scope of treatment modality measures consisted of frequency estimation of a wide range of techniques used in patient management: soft tissue, cervical spine high-velocity low-amplitude (HVLA), overall HVLA (HVLA applied overall, except to the cervical spine), cranial, visceral, cranio-sacral, fascial, biodynamic, functional, muscle energy techniques (MET), and reflex.

### Statistical analysis

Standard descriptive analyses (e.g., means and standard deviations for continuous variables and percentages for categorical variables) were used to summarize demographic variables, practice characteristics, patients’ profiles, scope of treatment modalities, health promotion, research, and osteopathic practice. Comparisons between subgroups of continuous variables were completed by using independent t-tests or nonparametric tests, depending on normality of distribution. Other associations were explored by using bivariate and multivariate analyses. Associations between the use of techniques and age, gender, years of practice, and GDK-CDS diploma were explored with multivariate logistic regression. In particular, for each technique, the dependent variable was considered positive if the technique was used “often” or “very often” by the practitioner and negative if it was used “never,” “rarely,” or “sometimes.” The four independent variables were all dichotomized: <40 years old versus ≥40 (age), male versus female (gender), <15 years of practice versus ≥15, and GDK-CDS diploma versus no GDK-CDS diploma. Analyses were performed with R (3.4.3) statistical software.

## Results

### Sociodemographic data

Among the 838 osteopaths who received the link to the questionnaire, 241 completed it, for an overall response rate of 28.8%. Almost two thirds of osteopaths were female. Ages ranged from 25 to 72 years with an overall mean of 42.0 (SD 10.7) years. Female practitioners were younger (mean age 38.5; SD 9.4) than male practitioners (mean age 47.9; SD 11.6). The socio-demographic data are reported in Table 1.

**Table 1:** Socio-demographic data of osteopath respondents (N = 241).

	n (%) or mean [SD]
***Gender (n = 239)***	
**Female**	150 (62.8)
**Male**	89 (37.2)
***Age (y*.*o*.) *(n = 235)***	
**≤35**	104 (24.1)
**35–49**	166 (38.4)
**50–64**	139 (32.2)
**≥65**	23 (5.3)
**Mean age of**	
GDK-CDS^a^ diploma holders (n = 193)	41.9 [SD 10.2]
assistants (n = 25)	30.6 [SD 4.8]
Non-GDK-CDS diploma holders (n = 23)	54.8 [SD 10.5]
***Training country (n = 241)***	
**Switzerland**	169 (70.1)
**France**	53 (22.0)
**England**	9 (3.75)
**Elsewhere in Europe**	9 (3.75)
**Canada**	1 (0.4)
***Years of practice (n* = *240)***	
**<5**	56 (12.6)
**5–9**	118 (26.5)
**10–14**	98 (22.0)
**≥15**	173 (38.9)
**Mean**	12.7 [SD 7.6]
***GDK-CDS diploma (n = 239)***	
**Yes**	193 (80.8)
No^b^	46 (19.2)

^a^GDK-CDS: Swiss Conference of Cantonal Health Directors

^b^Of whom 54.3% (n = 25) were in an assistantship

### Practice characteristics

The majority of osteopaths were self-employed (80.8%, n = 193). Most osteopaths worked in one (72.5%, n = 174) or two (24.1%, n = 58) practice locations. A majority of osteopaths worked exclusively in a group practice (72.6%, n = 175) and 22.4% (n = 54) worked exclusively alone. The mean number of monthly consultations was 117.3 (SD 50.8). The average weekly number of hours of practice was 34 (SD 10.3), including 3.5 hours dedicated to administrative paperwork related to clinical practice. On average, the respondents took 5.7 weeks (SD 1.8) of vacation per year. The mean time spent with their patients was 48.8 minutes (SD 7.5) on the first visit, 45.0 minutes (SD 6.9) for a return visit, and 42.4 minutes (SD 7.3) for a follow-up visit.

Over one fourth (27.8%, n = 65) of osteopaths could arrange an appointment for an acute condition on the same day, and 62.0% could do so within a week. Regarding nonurgent appointments, 3.8% (n = 9) of osteopaths could arrange a nonurgent appointment the same day, 48.2% (n = 113) the following week, and 44.0% the following month (n = 103).

### Patient profiles

The participants reported that the majority (54.6%, n = 130) of patients were 19–64 years old, 14.1% (n = 33) were 2 years old or younger, 15.9% (n = 37) were between 3 and 18 years old, and 16.0% (n = 38) were older than 65 years. The mean proportion of female patients reported by osteopaths was 63.4% (SD 9.0).

**Fig 1:**
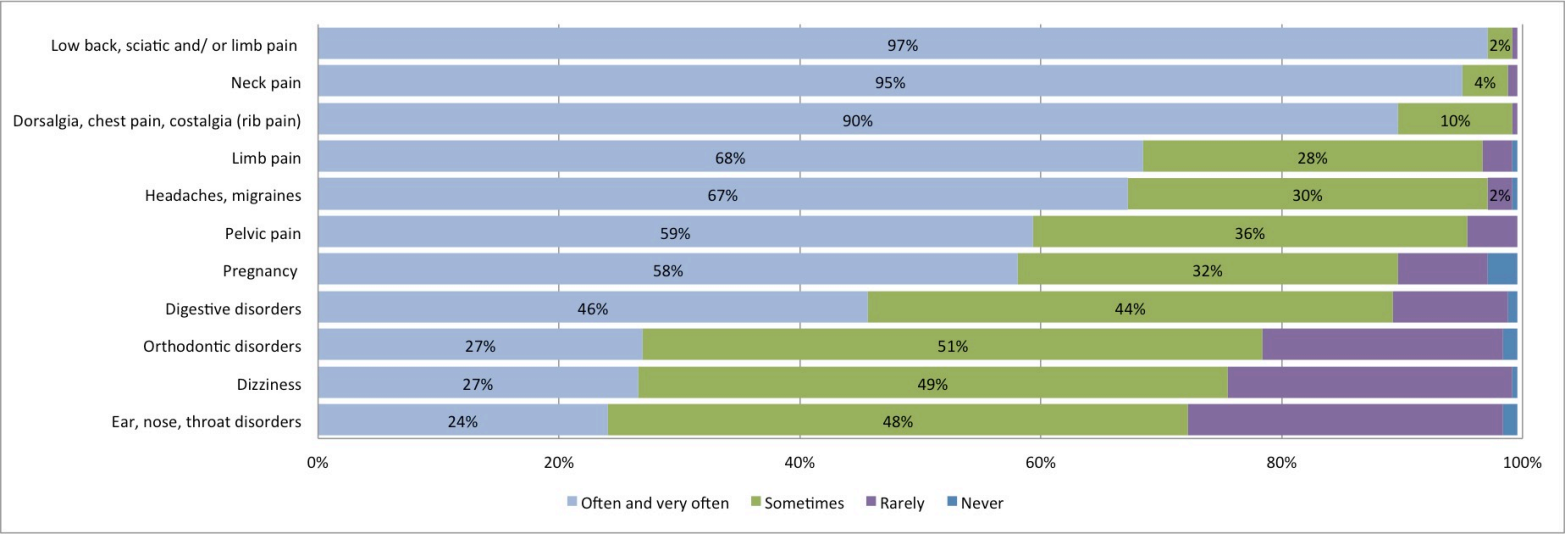
Frequency of primary reasons for consultation for adult patients (≥18 years) during the past month, as reported by osteopaths.

**Fig 2:**
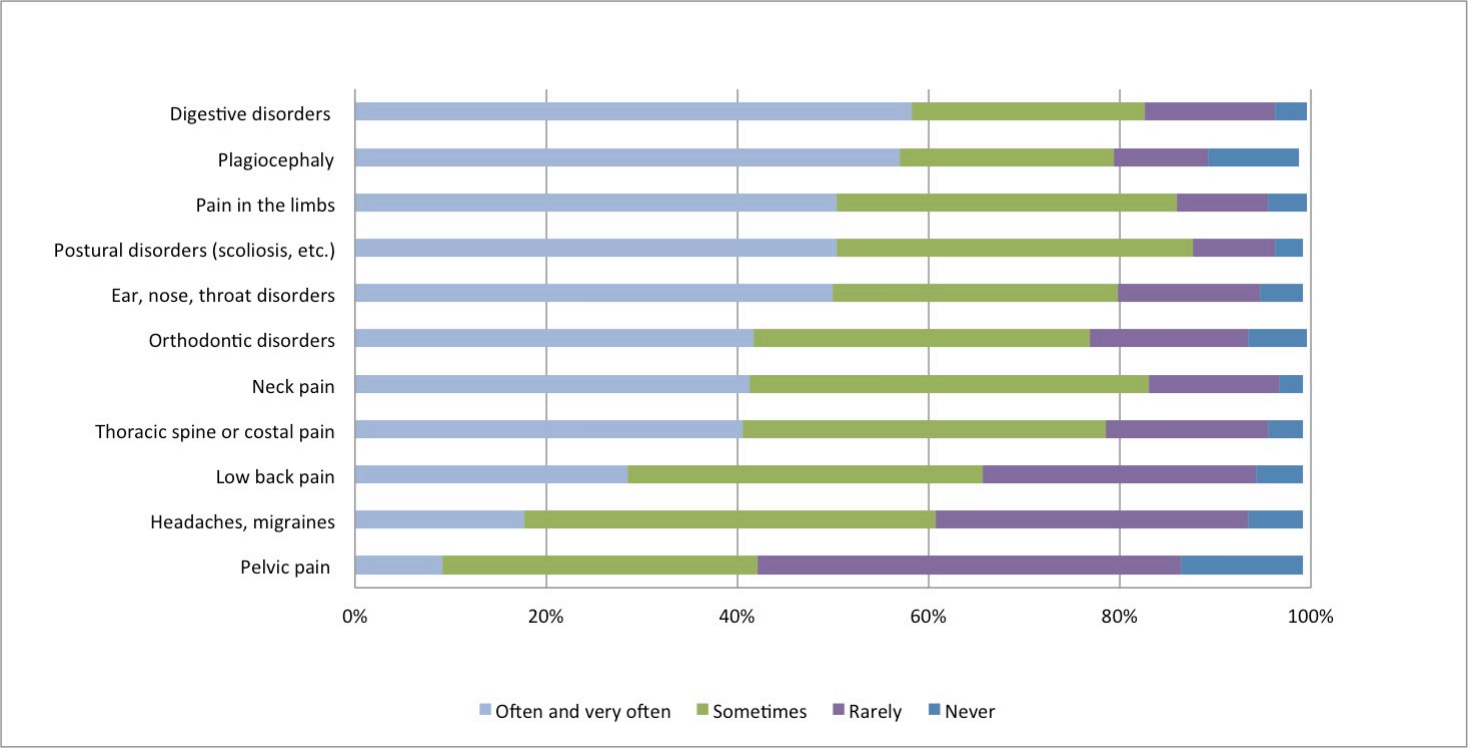
Frequency of primary reasons for consultation for pediatric patients (<18 years) during the past month, as reported by osteopaths.

The top three reasons for consultation reported by osteopaths in the past year, rated as often and very often, were low back pain (including sciatic pain and lower limb pain), followed by neck pain and then thoracic spine pain (including thoracic pain and rib pain). The frequencies of the primary reported reasons for consultation for adults are presented in Fig 1. For pediatric patients (<18 years old), the top three reported reasons for consultation in the past year, rated as often and very often, were digestive disorders, plagiocephaly, and a tie between limb pain and postural disorders. The frequency of the primary reported reasons for consultation for pediatric patients are presented in Fig 2.

Respondents reported that patients consulted mainly for pain that lasted less than four weeks (acute) and for pain that lasted four to six weeks (subacute) (n = 111, 47.0% and n = 80, 33.3%, respectively), followed by patients who consulted for chronic pain (n = 50, 21.2%).

### Number of consultations provided for acute low back and neck pain management

For the management of acute low back pain and acute neck pain, 94.4% of responding osteopaths estimated that one to three treatments were required for the management of those symptoms. More precisely, for acute low back pain, 7.7% (n = 18) of participants estimated improvement or resolution to be achievable in one visit, 44.4% (n = 104) in two visits, 42.7% (n = 100) in three visits, and 4.7% (n = 11) in four to six visits. For acute neck pain, 7.7% (n = 18) of participants estimated improvement or resolution to be achievable in one visit, 48.7% (n = 114) in two visits, 38.0% (n = 89) in three visits, and 5.6% (n = 13) in four to six visits. More than 80% (n = 182) of osteopaths considered that every second patient with acute low back pain consulted them exclusively.

### Scope of treatment

The frequency of techniques used in osteopaths’ daily practice is presented in Table 2. Visceral treatment and soft tissue techniques were the most often used. Almost half of osteopaths never used biodynamic techniques and more than a quarter never used cervical spine HVLA thrust techniques. Overall, osteopaths’ clinical practice encompassed a wide variety of techniques.

**Table 2:** Reported frequency of different techniques used by osteopaths.

Frequency				
Treatment modality	Often/very often	Sometimes	Rarely	Never
Visceral (n = 228)	216 (89.6)	18 (7.5)	3 (1.2)	0 (0)
Soft tissue (n = 228)	192 (79.7)	31 (12.9)	11 (4.6)	3 (1.2)
Muscle energy technique (n = 228)	183 (75.9)	38 (15.8)	12 (5)	4 (1.7)
Cranial (n = 228)	181 (75.1)	48 (19.9)	7 (2.9)	1 (0.4)
Functional (n = 227)	173 (71.8)	37 (15.4)	18 (7.5)	8 (3.3)
Fascial (n = 226)	162 (67.3)	41 (17)	24 (10)	8 (3.3)
Overall HVLA (n = 227)	132 (54.8)	47 (19.5)	36 (14.9)	21 (8.7)
Cranio-sacral (n = 226)	112 (46.4)	55 (22.8)	28 (11.6)	41 (17)
Cervical spine HVLA (n = 224)	68 (28.2)	41 (17)	61 (25.3)	63 (26.1)
Reflex (n = 228)	63 (26.1)	72 (29.9)	67 (27.8)	35 (14.5)
Biodynamic (n = 214)	51 (21.2)	32 (13.3)	25 (10.4)	115 (47.7)

Results are reported as n (%)

HVLA: high-velocity low-amplitude technique

Cervical spine HVLA: High-velocity low-amplitude (HVLA) techniques applied to the cervical spine.

Overall HVLA: HVLA techniques applied overall (except to the cervical spine)

Definitions of the different treatment modalities can be found in the *Glossary of Osteopathic Terminology* [28]

### Health promotion and integration

All osteopaths reported discussing health promotion and disease prevention in areas such as postural hygiene and physical activity (95.0%, n = 229), depression (52.5%, n = 127), lifestyle habits and nutrition (48.6%, n = 117), smoking prevention (37.8%, n = 91), melanoma (24.9%, n = 60), alcohol prevention (24.5%, n = 59), and breast cancer (19.1%, n = 46) as part of their patients’ management. A total of 94.7% (n = 216) of participants felt that osteopathy should be integrated into usual care. More than half of the participants (54.2%, n = 129) were not in favor of having consultation costs covered by basic health insurance, 27.7% (n = 66) were in favor, and 18.1% (n = 43) answered that they did not know.

### Research

Almost all participants (98.7%, n = 226) were in favor of research regarding osteopathy. Participants evaluated the importance (ranging from extremely important to not important at all) of eight research fields. More than two thirds of the participants estimated that the following research topics ranged from important to extremely important: defining the role of osteopathy within the healthcare system (68.0%, n = 153) and studying the efficacy of osteopathic treatments (67.1%, n = 153), the mechanism of action (63.7%, n = 142), and the risks (61.4%) of osteopathic treatment. Half of the participants (52.3%, n = 113) felt that research on the cost-effectiveness of osteopathic management was important to extremely important. Less than a third of participants felt that describing practitioners’ profiles (27.6%, n = 55) and patients’ profiles (29.4%, n = 58) was important to extremely important.

### Correlates of osteopathic practice

In our sample, the mean number of years of practice was higher in male osteopaths than in female osteopaths (males: 16.2 years [SD 8.2], females: 10.5 years [SD 6.2], p<0.001), as were the weekly working hours (males: 38.2 [SD 11.0], females: 31.6 [SD 8.9], p<0.001). On average, male osteopaths reported more monthly consultations than female osteopaths did (139.1 [SD 63.5] vs 105.0 [SD 37.1], respectively, p<0.001), as well as seeing more new patients per month (23.2 [SD 20.1] vs 16.5 [SD 12.6], respectively, p<0.01). Female osteopaths reported spending more time during an encounter with a new patient than male osteopaths did (females: 50.0 minutes [SD 6.9], males: 46.6 [SD 8.0], p<0.001), at a follow-up visit (females: 44.3 [SD: 6.2], males: 39.5 [SD 8.0], p<0.001), and for a returning patient and a new reason for consultation (females: 46.5 [SD 6.0], males: 42.8 [SD 7.5], p<0.001).

In multivariate logistic regression analyses, we explored each technique used by respondents in their daily practice adjusted for age, gender, years in practice, and having a GDK-CDS diploma (Table 3). Older age was positively associated with the use of biodynamic and reflex techniques. Being a female was negatively associated with daily use of HVLA thrust techniques for the cervical spine and overall HVLA, whereas being female was positively associated with cranial, cranio-sacral, biodynamic, and fascial techniques. Practicing for ≥15 years was negatively associated with the use of overall HVLA techniques. Having a GDK-CDS diploma was positively associated with the use of visceral techniques.

**Table 3:** Multivariate logistic regression analysis of each technique used by osteopaths in their daily practice, adjusted for age, gender, years of practice, and GDK-CDS diploma.

Treatment modality	Age	Gender	Years of practice	GDK-CDS diploma
	(<40^a^/ ≥40)	(male^a^/female)	(<15^a^/ ≥15)	(yes^a^/no)
	OR (95% CI)	OR (95% CI)	OR (95% CI)	OR (95% CI)
Cervical spine HVLA (n = 224)	0.8 (0.3–1.9)	0.3 (0.2–0.5)***	0.8 (0.3–2.3)	0.7 (0.3–1.5)
Overall HVLA (n = 227)	0.7 (0.3–1.8)	0.3 (0.1–0.5)***	0.4 (0.2–1.0)*	1.3 (0.7–2.7)
Functional (n = 227)	0.8 (0.3–2.0)	1.2 (0.6–2.3)	1.3 (0.5–3.4)	0.5 (0.2–1.1)
Muscle energy technique (n = 228)	1.0 (0.4–3.0)	1.0 (0.5–2.1.0)	0.5 (0.2–1.4)	1.3 (0.6–2.9)
Visceral (n = 228)	0.3 (0.1–1.8)	1.6 (0.6–4.3)	0.6 (0.1–2.0)	3.3 (1.1–9.4)**
Cranial (n = 228)	1.1 (0.4–3.1)	2.7 (1.4–5.4)**	1.2 (0.4–3.1)	1.9 (0.9–4)
Soft tissue (n = 228)	2.9 (0.9–13.8)	1.8 (0.9–3.8)	0.4 (0.1–1.3)	0.9 (0.3–2.1)
Fascial (n = 226)	1.1 (0.4–2.8)	1.9 (1.0–3.5)*	1.0 (0.4–2.5)	1.0 (0.4–2.0)
Biodynamic (n = 214)	4.6 (1.7–12.4)**	2.2 (1.0–4.8)*	0.7 (0.3–1.9)	0.8 (0.3–1.8)
Cranio-sacral (n = 226)	2.0 (0.9–4.8)	2.1 (1.1–3.9)*	1.1 (0.5–2.6)	0.9 (0.4–1.8)
Reflex (n = 228)	3.1 (1.2–7.7)*	0.7 (0.4–1.3)	0.8 (0.3–1.9)	1.4 (0.6–3.4)

* p-value <0.05

** p-value <0.01

*** p-value <0.001

^a^Reference group

OR: odds ratio, CI: confidence interval, HVLA: high-velocity low-amplitude techniques, impulse manipulations

Cervical spine HVLA: High-velocity low-amplitude (HVLA) techniques applied to the cervical spine.

Overall HVLA: HVLA techniques applied overall (except to the cervical spine)

Definitions of the different treatment modalities can be found in the *Glossary of Osteopathic Terminology [28]*

## Discussion

Our results provide insights into osteopaths’ profiles and practice activities with an emphasis on their management of patients with back pain. In this study, osteopaths mostly treated patients with acute symptoms, primarily spinal pain, and most of the time, one to three consultations were reported to manage such conditions. Visceral and soft tissue techniques were the most frequently applied, whereas biodynamic and cervical HVLA thrust techniques were less frequently used.

Regarding clinical practice characteristics, the mean number of years of osteopathic practice was higher for males than for females, as were weekly working hours. The higher number of working hours for male osteopaths could be explained by more male osteopaths working full time, a gender gap found in other professions [29]. As for the number of years of practice, the difference between males and females could be linked with an increasing number of women embracing the profession of osteopathy. Considering that osteopathy has been regulated for only a few years in Switzerland, data are not yet available on that matter. Moreover, similar to male primary care physicians [30], male osteopaths in our sample reported on average more monthly consultations and saw more new patients than females did. As described previously, the majority of osteopaths were self-employed and working in private group practices [15, 16]. The results regarding osteopaths’ consultation length and time spent with the patient at the first visit, the return visit, and the follow-up visit were very close to previous results [11, 15]. Moreover, female osteopaths reported spending more time during an encounter with their patients, as described previously for female primary care physicians [31–33]. Prior to this study, a Swiss study provided an overview of osteopaths across Switzerland [16], which supports the results of our sociodemographic and clinical practice characteristics. Indeed, both studies demonstrated a similar proportion of male and female osteopaths, as well as a similar number of years of practice and a predominant trend in self-employment. Moreover, the main location of complaints was also comparable (spinal area).

Concerning the techniques used, our results present many similarities with comparable studies conducted in Switzerland, Canada, Australia, and the United Kingdom [10, 11, 13, 16,34]. For example, visceral techniques were also the most frequently used in the Benelux [15], and soft tissue techniques were the most used among UK, Australian, and Swiss osteopaths [10, 13, 16, 34]. Visceral techniques cover a broad spectrum of therapeutic indications, which would explain their high prevalence in some countries. However, there is no current evidence of the efficacy of such techniques{Guillaud, 2018 #697}. In addition, older age was positively associated with the use of biodynamic and reflex techniques. These findings could reflect changes in the training of osteopaths or could mirror a possible paradigm shift of the profession toward more evidence-based techniques. Such a shift has already been observed among osteopathic physicians in the United States [35].

Cervical spine HVLA techniques (thrust techniques) were the second least frequently used method. Indeed, cervical HVLA manipulation is a source of much debate in manipulative therapy because of the potential serious adverse events related to this technique [36, 37]. Lesser use of HVLA techniques could thus also mirror a lack of confidence in using them. Being a female osteopath was negatively associated with the use of cervical spine HVLA and overall HVLA. Differences in practice patterns between genders has recently been described in medical settings in which female physicians showed fewer risk-taking behaviors [38, 39]. This could also be the case with our respondents regarding cervical HVLA spinal manipulations.

In this study, osteopaths reported mostly treating patients with acute symptoms, which were primarily spinal conditions, mainly low back and neck pain, as confirmed by a recent Swiss study [16] and other international studies [10, 11, 16, 40]. Cost-effectiveness studies showed encouraging results [41–43] in the use of osteopathy in acute settings. However, regarding the small number of consultations scheduled by our respondents for acute low back pain and neck pain management, further studies should explore whether these prompt management capabilities provide a clinically meaningful difference for pain or disability in people with LBP when compared with the natural course of back pain [44, 45].

Regarding the primary reason for pediatric consultations reported by our respondents, a Canadian study described similar findings, consultations being mostly for plagiocephaly disorders, postnatal torticollis assessment, and otorhinolaryngeal disorders [11].

Osteopaths rated research in osteopathy as important, which is in line with previous results [11, 15], especially in areas such as describing the role of osteopathy within the healthcare system and studying the efficacy, mechanism of action, and risks of osteopathic treatments. This finding reflects an existing interest of the profession toward evidence-based practice. Further studies should address osteopaths’ perception of research for osteopathic practice, as has been done in previous studies [46].

Although almost all participants considered that osteopathy should be integrated into the conventional care system, the majority also stated that they did not want to have their consultation costs covered by the mandatory basic health insurance. However, in order to be fully integrated in the conventional care system and accessible to the whole Swiss population, osteopathy would have to be part of the mandatory health insurance coverage. This discrepancy might reflect concerns of our respondents regarding loss of professional identity and freedom due to restrictions on practice, as well as increased paperwork. Notwithstanding this, considering the high use of osteopathy for back pain only and the increasing data on the benefits of osteopathy for back pain [7, 47–50], as well as the short waiting time for an appointment reported by our respondents, reimbursement by basic health insurance should be further considered by osteopaths and policy makers. As shown in several studies, emergency departments are increasingly filled with non-life-threatening conditions such as spinal pain [51–55]. In this context, osteopathy could constitute an added value for emergency facilities. Osteopathy could thus help to reduce unscheduled treatment use in the secondary care sector and should be taken into account for the primary care management of spinal conditions. Further research should explore this question, especially the cost-effectiveness of osteopathy [41, 56].

### Limitations

The study has some limitations. The questionnaire was not validated, which has implications on the reliability and the internal consistency of the information collected. In addition, the response rate was relatively low and female osteopaths were overrepresented in our study compared with the number of female osteopaths in the registries (62.8% vs 51.6%); thus, our results might not be generalizable to the entire osteopathic population. Our sample was otherwise representative in terms of age and practice location. A lack of interest in this type of research could explain the low response rate, since only less than a third of participants considered that describing practitioners’ profiles and patients’ profiles was important to extremely important. The length of the questionnaire may also have negatively influenced the response rate. Second, because the questionnaires were collected during a specific three-month period, it is possible that our findings are not representative of an annual consultation. Third, the questionnaire was self-administered which could induce a social desirability bias. Finally, we surveyed only osteopaths who were working in the French-speaking part of Switzerland, as logistical constraints did not allow us to carry out a survey on the whole of Switzerland in two other languages (German and Italian).

## Conclusion

Our study provides new information about the characteristics of osteopaths in the French-speaking part of Switzerland, particularly about their role in the management of spinal conditions. These findings, combined with short waiting times for consultations for acute conditions, as well as prompt management capabilities for acute low back and acute neck pain, support the premise that the osteopathic profession could constitute an added value to primary healthcare.

## Supporting information

S1 Survey Osteopathy in the French-speaking part of Switzerland. Practitioners’ profile and scope of back pain management (an online survey).
(DOCX)

S2 Survey L'ostéopathie en suisse romande. Profil des praticiens et prise en charge du mal de dos (un questionnaire en ligne).
(DOCX)
