# Supplementary material for: Osteopathy in the French-speaking part of Switzerland: Practitioners’ profile and scope of back pain management
Source: PLoS One. 2020 May 1;15(5):e0232607. doi: 10.1371/journal.pone.0232607 (PMC7194435; doi:10.1371/journal.pone.0232607)
Supplement: S1 Survey — Practitioners’ profile and scope of back pain management (an online survey). (DOCX) [file pone.0232607.s001.docx]

## Osteopathy in the French-speaking part of Switzerland: practitioners’ profile and scope of back pain management *an online survey*

## NB: questions marked with * are mandatory

# Introduction

1. In which canton do you practice?* (Please select an answer below)

- Berne
- Fribourg
- Genève
- Jura
- Valais
- Vaud
- Neuchâtel
- I do not wish to answer
- Other

🖂 If you practice in more than one canton, please indicate the canton where you mainly practice.

# Socio-demographic data

1. What is your gender?* (Please select an answer below)

- Male
- Female
- I do not wish to answer

1. In what year were you born?* (Only numbers can be entered in this field)

🖂 Please enter the 4 digits of your year of birth.

If you do not wish to answer, please enter 333.

1. What is your nationality? (Please select an answer below)

- Swiss
- Other
- No response

🖂 If you are of another nationality, please specify which one.

1. What is the postal code of the area where your practice is located? (Only numbers can be entered in this field)

Postal code

🖂 Please enter the 4 digits of your postal code. This question is intended to map the distribution of practices between urban, peri-urban, and rural areas and will not identify you. If you have more than one practice, please indicate the postal code of your main practice.

# Training

1. In what year did you receive your diploma in osteopathy (DO)?*

🖂 If you do not have a D.O., please enter 222.
🖂 If you do not wish to answer, please enter 333.

1. In which country did you do your main training in osteopathy (DO)? (Please select an answer below)

- Switzerland
- France
- Germany
- England
- Other

🖂 If you completed your training in another country, please specify which one.

1. Do you have the CDS/GDK diploma? (Please select an answer below)

- Yes
- No
- I do not wish to answer

If Yes, in which year did you receive your CDS/GDK diploma? (Only numbers can be entered in this field)

If No, are you currently an assistant osteopath? (Please select an answer below)

- Yes
- No

Do you plan to take the CDS examination? (Please select an answer below)

- Yes
- No

If No, are you an etiopath? (Please select an answer below)

- Yes
- No

**4.** Do you have full training (excluding continuing education) in other method(s) of complementary medicine? (Please select an answer below)

- Yes
- No

If Yes, in which method(s)?

🖂 If you have completed training in several methods, please list them all

If Yes, do you currently practice this/these other method(s)? (Please select an answer below)

- Yes
- No

🖂 If you have full training in several other methods but are currently practicing only one, please answer yes.

**5.** Do you have a degree in Western conventional medicine?*

- Yes
- No
- I do not wish to answer

**6.** Did you learn any other profession apart from complementary medicine?* (Please select an answer below)

- Yes
- No

If Yes, which profession(s)?

🖂 If you have more than one, please list them all

If Yes, please specify (Please select an answer below)

- I still practice this profession
- I used to practice this profession but I don't practice it anymore
- I never practiced this profession

# Continuing education

1. Approximately how many hours of continuing education have you completed in the **last 12 months**? (Only numbers can be entered in this field)

1. In general, what kind of continuing education do you do? (Check all that apply)

- Practical/theoretical training in your current profession
- Practical/theoretical training in in another health-related field
- Participation in symposia/congresses/conferences
- Participation in case discussion/colloquiums
- Reading scientific articles
- Other

# Practice

1. How many years have you been practicing osteopathy?* (Only numbers can be entered in this field)

years

🖂 If you have been practicing for less than a year, please enter a zero.

If you do not wish to reply, please enter 333.

1. Is the practice of osteopathy your **main professional** activity? *****(Please select an answer below)

- Yes
- No
- I share my professional activity equally between two professions
- I do not wish to answer

1. On average, how many hours per week do you practice osteopathy (including paperwork)?* (Only numbers can be entered in this field)

hours per week

🖂 If you do not wish to reply, please enter 333.

1. On average, how many hours per week do you spend on professional administrative tasks related to osteopathy (administration, correspondence, insurance, etc.)? (Only numbers can be entered in these fields)

hour(s) minutes

🖂 If you spend less than an hour, please enter 0 in the hours box and the number of minutes in the minutes box.

1. Generally, how quickly can a patient get an **urgent** appointment with you? (Please select an answer below)

- On the same day
- 2 to 7 days (within a week)
- 8 to 30 days (within a month)
- Beyond 30 days
- No answer

1. Generally, how quickly can a patient get a **non-urgent appointment** with you? (Please select an answer below)

- On the same day
- 2 to 7 days (within a week)
- 8 to 30 days (within a month)
- Beyond 30 days
- No answer

1. In general, how many weeks of **vacation per year** do you have? (Only numbers can be entered in this field)

weeks per year

1. Are you a member of a professional association/federation? (Please select an answer below)

- Yes
- No
- No answer

🖂 except for ASCA and RME

If No, why?

# Work environment

1. What is your professional status? (*Please select an answer below*)

- Self-employed therapist
- Employed therapist
- Both
- No answer

1. How many practices do you practice in? (Please select an answer below)

- None
- 1
- 2
- More than 2

1. Which situation best suits your work environment? (Check all that apply)

- I work alone in a practice
- I work in a group practice where we share only our premises
- I work in a group practice where there is collaboration between the different therapists in the practice
- I work in an osteopathic clinic
- Other

🖂 If you practice in more than one practice, please answer for each one.

1. Do you share your practice with other complementary medicine therapists (including osteopaths)? (Please select an answer below)

- Yes
- No
- No answer

If Yes, what is the profession of the **complementary medicine** therapists with whom you share your practice? (Check all that apply)

- Osteopath
- Assistant osteopath
- Acupuncturist
- Naturopath
- Massage therapist
- Other

1. Do you share your practice with **conventional medicine** therapists? (Please select an answer below)

- Yes
- No
- No answer

If Yes, what is the profession of the conventional medicine therapists with whom you share your practice? (Check all that apply)

- Medical doctor
- Physiotherapist
- Dentist
- Midwife
- Other

# Patients

1. On average, how many visits do you make per month?* (Only numbers can be entered in this field)

🖂If you do not wish to answer, please enter 333.

1. On average, how many new patients do you treat per month? (Only numbers can be entered in this field)

🖂 If you do not know, please enter 333.

1. On average, how many minutes does one of your consultations last? (Only numbers can be entered in these fields)

| For a new patient |  |
| --- | --- |
|  |  |
| For a former patient with a new reason for consultation |  |
|  |  |
| For a follow-up |  |

1. For the same patient, how many consultations do you consider necessary, on average, for the following reasons for consultation? *(Only numbers can be entered in these fields)*

| Acute low back pain (one episode) |  |
| --- | --- |
| Acute neck pain (one episode) |  |
| Chronic low back pain (number of appointments per year) |  |
| Chronic neck pain (number of appointments per year) |  |

1. According to your estimate, what is the percentage of **women** in your patient population? (Only numbers can be entered in this field)

%

1. Overall, what percentage of your patients are in the following age groups? (Only numbers can be entered in these fields)

| Children 0-2 years old |  |
| --- | --- |
| Children 3-18 years old |  |
| Adults 19-64 years old |  |
| Adults 65 years old and over |  |

🖂 Please give a percentage estimate for each age group. Ideally they should total 100%.

1. Have you specialized your practice in any of the following areas? (Check all that apply)

- No, I did not specialize my practice in any particular area
- Pediatric and/or obstetric osteopathy
- Gynecological and/or perineal osteopathy
- Sports osteopathy
- Geriatric osteopathy
- Visceral osteopathy
- Cranial osteopathy
- Biodynamic osteopathy
- Cranio-sacral osteopathy
- Other: ______________________________________________

1. How often do you use the following techniques?*

|  | Never | Seldom | Sometimes | Often | Very often | I do not wish to answer |
| --- | --- | --- | --- | --- | --- | --- |
| High-velocity low-amplitude (HVLA) techniques applied to the cervical spine |  |  |  |  |  |  |
| HVLA techniques applied overall (apart from cervical) |  |  |  |  |  |  |
| Functional techniques |  |  |  |  |  |  |
| Muscle energy techniques |  |  |  |  |  |  |
| Visceral osteopathy |  |  |  |  |  |  |
| Cranial osteopathy |  |  |  |  |  |  |
| Soft tissue techniques |  |  |  |  |  |  |
| Fascial techniques |  |  |  |  |  |  |
| Biodynamic techniques |  |  |  |  |  |  |
| Cranio-sacral osteopathy |  |  |  |  |  |  |
| Reflex technique |  |  |  |  |  |  |

1. Do you practice any other techniques? (Please select an answer below)

- Yes
- No

If Yes, which techniques?

1. In your opinion, what percentage of your patients have supplementary health insurance that reimburse them for your care? (Only numbers can be entered in this field)

%

1. In your opinion, what percentage of your patients consult you for a problem covered by the Federal Law on Accident Insurance? (LAA)? *(Only numbers can be entered in this field)*  %

# Main reasons for consultations

1. What are the five most frequent reasons your patients give for coming to see you (the five main reasons for consulting)?

| Main reason N°1 |  |
| --- | --- |
| Main reason N°2 |  |
| Main reason N°3 |  |
| Main reason N°4 |  |
| Main reason N°5 |  |

🖂 *Please list them by indicating the most frequent reason for consultation first.*

1. On average, in the last month, how often did your patients visit for the following main reasons?

| Reason for consultation | Never | Seldom | Sometimes | Often | Very often |
| --- | --- | --- | --- | --- | --- |
| Osteopathic checkup (prevention or control) |  |  |  |  |  |
| Acute pain (0-4 weeks) |  |  |  |  |  |
| Subacute pain (4-12 weeks) |  |  |  |  |  |
| Chronic pain (over 12 weeks) |  |  |  |  |  |
| Decreased mobility (pain-free) |  |  |  |  |  |

1. In your opinion, what percentage of your patients with acute low back pain see you exclusively (without the intervention of another health professional)? (Only numbers can be entered in this field)

%

1. In general, over the past year, how often have your adult patients visited you for the following main reasons?

|  | Never | Seldom | Sometimes | Often | Very often | I do not wish to answer |
| --- | --- | --- | --- | --- | --- | --- |
| Headaches, migraines |  |  |  |  |  |  |
| Neck pain |  |  |  |  |  |  |
| Dorsalgia, chest pain, costal pain |  |  |  |  |  |  |
| Low back pain, sciatica, cruralgia |  |  |  |  |  |  |
| Pelvic pain (pubalgia, sacralgia, etc.) |  |  |  |  |  |  |
| Pain in the limbs |  |  |  |  |  |  |
| Digestive disorders |  |  |  |  |  |  |
| Vertigo |  |  |  |  |  |  |
| ENT sphere disorder |  |  |  |  |  |  |
| Orthodontic disorders (bruxism, malocclusion, etc.) |  |  |  |  |  |  |
| Pregnancy follow-up |  |  |  |  |  |  |

1. In general, in the past year, how often have your pediatric patients (0-18 years) visited you for the following main reasons?

|  | Never | Seldom | Sometimes | Often | Very often | I do not wish to answer |
| --- | --- | --- | --- | --- | --- | --- |
| Plagiocephaly |  |  |  |  |  |  |
| Headaches, migraines |  |  |  |  |  |  |
| Neck pain |  |  |  |  |  |  |
| Dorsalgia, chest pain, costalgia |  |  |  |  |  |  |
| Low back pain, sciatica |  |  |  |  |  |  |
| Pelvic pain (pubalgia, sacralgia, etc.) |  |  |  |  |  |  |
| Pain in the limbs |  |  |  |  |  |  |
| Posture disorders (scoliosis, etc.) |  |  |  |  |  |  |
| Digestive disorders (GERD, colic, constipation, etc.) |  |  |  |  |  |  |
| ENT disorders (otitis, angina, sucking, swallowing, etc.) |  |  |  |  |  |  |
| Orthodontic disorders (bruxism, malocclusion, etc.) |  |  |  |  |  |  |

# Patient education

1. What prevention theme(s) do you usually address in consultation? (Check all that apply)

- None
- Postural hygiene
- Tobacco
- Alcohol problem
- Melanoma
- Depression
- Breast cancer
- Obesity
- Other

🖂 If Other, please specify.

# Scientific research

1. Are you in favor of osteopathic research? (Please select an answer below)

- Yes
- No

1. In your opinion, how important is it for the research themes below to be addressed in osteopathy?

1=Extremely important 5=Not important at all

| Definition of patient profile | 1 | 2 | 3 | 4 | 5 |
| --- | --- | --- | --- | --- | --- |
| Definition of therapist profile | 1 | 2 | 3 | 4 | 5 |
| Definition of the role of osteopathy within the healthcare system | 1 | 2 | 3 | 4 | 5 |
| Treatment effectiveness studies | 1 | 2 | 3 | 4 | 5 |
| Treatment-related risk studies | 1 | 2 | 3 | 4 | 5 |
| Studies on treatment side effects | 1 | 2 | 3 | 4 | 5 |
| Studies on treatment mechanisms of action | 1 | 2 | 3 | 4 | 5 |
| Treatment cost-effectiveness studies | 1 | 2 | 3 | 4 | 5 |

1. Would you like to contribute to osteopathic research? (Please select an answer below)

- Yes
- No

1. How would you like to contribute to osteopathic research? (Check all that apply)

- By participating in research conducted in the practice with patients
- By answering questionnaires
- By providing anonymous data on patient populations
- I do not wish to answer

# Place in the healthcare system

1. Do you think that osteopathy should be covered by the basic mandatory health insurance (LAMal)? (Please select an answer below)

- No
- Somewhat no
- Somewhat yes
- Yes
- I don't know

1. Do you think an osteopathy consultation should be offered in a hospital setting? (Please select an answer below)

- Yes
- No

If Yes, in which hospital sector do you think osteopathy should be available as a priority?

| **Proposed sector** |  | **Your ranking** |
| --- | --- | --- |
| Emergency |  |  |
| Rheumatology |  |  |
| Oncology |  |  |
| Internal Medicine |  |  |
| Psychiatry |  |  |
| Neurology |  |  |
| Gynecology/Obstetrics |  |  |
| Pediatrics |  |  |
| Dermatology |  |  |
| Traumatology/ Orthopedics |  |  |
| Surgery |  |  |

Please rank the above areas in order of priority, starting with the highest. Please rank at least five.
