# Supplementary material for: Osteopathy in the French-speaking part of Switzerland: Practitioners’ profile and scope of back pain management
Source: PLoS One. 2020 May 1;15(5):e0232607. doi: 10.1371/journal.pone.0232607 (PMC7194435; doi:10.1371/journal.pone.0232607)
Supplement: S2 Survey — Profil des praticiens et prise en charge du mal de dos (un questionnaire en ligne). (DOCX) [file pone.0232607.s002.docx]

**L'ostéopathie en suisse romande : profil des praticiens et prise en charge du mal de dos**

*Un questionnaire en ligne*

NB : Les questions avec un astérisque (*) sont obligatoires.

# Introduction

1. Dans quel canton exercez vous ?* (Veuillez sélectionner une réponse ci-dessous)

- Berne
- Fribourg
- Genève
- Jura
- Valais
- Vaud
- Neuchâtel
- Je ne souhaite pas répondre
- Autre

🖂Si vous exercez dans plusieurs cantons, merci d'indiquer celui où vous exercez principalement

# Données socio-démographiques

1. Quel est votre genre ? *(Veuillez sélectionner une réponse ci-dessous)

- Masculin
- Féminin
- Je ne souhaite pas répondre

1. Quelle est votre année de naissance ?* (Seuls des nombres peuvent être entrés dans ce champ)

🖂 Merci d’indiquer les 4 chiffres de votre année de naissance
Si vous ne souhaitez pas répondre, merci de noter 333.

1. Quelle est votre nationalité ? (Veuillez sélectionner une réponse ci-dessous)

- Suisse
- Autre
- Sans réponse

🖂 Si vous êtes d’une autre nationalité, merci de préciser laquelle

1. Quel est le code postal de la localité où se situe votre cabinet ? (Seuls des nombres peuvent être entrés dans ce champ)

Code postal

🖂 Merci d’indiquer les 4 chiffres de votre code postal. Cette question est destinée à établir une cartographie de la répartition des cabinets entre zone urbaine, péri-urbaine et rurale, et ne permettra pas de vous identifier. Si vous avez plusieurs cabinets, merci d’indiquer le code postal de votre cabinet principal.

# Formation

1. En quelle année avez-vous obtenu votre diplôme de fin d’étude en ostéopathie (DO)?*

🖂 Si vous n’avez pas de D.O, merci de noter 222
🖂 Si vous ne souhaitez pas répondre, merci de noter 333

En quelle année avez-vous terminé votre formation principale en naturopathie/acupuncture?* (Seuls des nombres peuvent être entrés dans ce champ)

🖂 Si vous ne souhaitez pas répondre, merci de noter 333

1. Dans quel pays avez-vous effectué votre formation **principale** en ostéopathie (DO)  ? (Veuillez sélectionner une réponse ci-dessous)

- Suisse
- France
- Allemagne
- Angleterre
- Autre :

🖂 Si vous avez effectué votre formation dans un autre pays, merci de préciser lequel

1. Avez-vous le diplôme CDS ?* (Veuillez sélectionner une réponse ci-dessous)

- Oui
- Non
- Je ne souhaite pas répondre

Si oui, en quelle année avez-vous obtenu votre diplôme CDS? (Seuls des nombres peuvent être entrés dans ce champ)

Si non, êtes-vous actuellement ostéopathe assistant ? (Veuillez sélectionner une réponse ci-dessous)

- Oui
- Non

Avez-vous l’intention de passer le CDS ? (Veuillez sélectionner une réponse ci-dessous)

- Oui
- Non

Si non, êtes vous étiopathes ? (Veuillez sélectionner une réponse ci-dessous)

- Oui
- Non

1. Avez-vous une formation complète (hors formation continue) dans une/des autre(s) méthode(s) de médecine complémentaire ? (Veuillez sélectionner une réponse ci-dessous)

- Oui
- Non

Si oui, dans quelle(s) méthode(s) ? :

🖂 Si vous avez des formations complètes dans plusieurs méthodes, merci de les lister en revenant à la ligne après chaque méthode

Si oui, pratiquez-vous **actuellement** cette/ces autre(s) méthode(s)? (Veuillez sélectionner une réponse ci-dessous)

- Oui
- Non

🖂 Si vous avez des formations complètes dans plusieurs autres méthodes mais n’en pratiquez qu’une actuellement, merci de répondre oui

1. Avez-vous un diplôme en médecine conventionnelle occidentale ?*

- Oui
- Non
- Je ne souhaite pas répondre

1. Avez-vous appris une autre profession en dehors du domaine des médecines complémentaires ?* (Veuillez sélectionner une réponse ci-dessous)

- Oui
- Non

Si oui, quelle(s) profession(s) ?

🖂 Si vous en avez plusieurs, merci de les lister toutes

Si oui, pouvez-vous préciser ? (Veuillez sélectionner une réponse ci-dessous)

- Je pratique encore cette profession
- J’ai pratiqué cette profession mais je ne la pratique plus actuellement
- Je n’ai jamais pratiqué cette profession

# Formation continue

1. Combien d’heures de formation continue environ avez-vous effectué durant les **12 derniers mois** ? (Seuls des nombres peuvent être entrés dans ce champ)

1. En général, quel type de formation continue effectuez-vous ? (Cochez la ou les réponses)

- Formation pratique/théorique dans votre profession actuelle
- Formation pratique/théorique dans un autre domaine de la santé
- Participation à des symposiums/congrès/conférences
- Participation à des colloques de discussion de cas
- Lecture d’articles scientifiques
- Autre

# Pratique

1. Depuis combien d’années pratiquez-vous l’ostéopathie ?* (Seuls des nombres peuvent être entrés dans ce champ)

années

🖂 Si vous pratiquez depuis moins d’une année, merci de mettre un zéro

Si vous ne souhaitez pas répondre merci de mettre 333.

1. La pratique de l’ostéopathie constitue-t-elle votre **activité professionnelle principale ? ***(Veuillez sélectionner une réponse ci-dessous)

- Oui
- Non
- Je partage à part égale mon activité professionnelle entre deux professions
- Je ne souhaite pas répondre

1. En moyenne, **combien d’heures par semaine** exercez-vous l’ostéopathie (y compris le travail administratif) ?* (Seuls des nombres peuvent être entrés dans ce champ)

heures par semaine

🖂 Si vous ne souhaitez pas répondre merci de mettre 333.

1. En moyenne, **combien d’heures par semaine** consacrez-vous aux tâches **administratives professionnelles** liées à l’ostéopathie  (administration, correspondance, assurances, etc…) ? (Seuls les nombres sont acceptés)

heure(s) minutes

🖂 Si vous y consacrez moins d’une heure, merci d’indiquer 0 dans la case des heures et n’indiquez que le nombre de minutes.

1. En général, dans quel délai un patient peut-il obtenir un rendez-vous **en urgence** avec vous ? (Veuillez sélectionner une réponse ci-dessous)

- Le jour même
- 2 à 7 jours (dans la semaine)
- 8 à 30 jours (dans le mois)
- Au delà de 30 jours
- Sans réponse

1. En général, dans quel délai un patient peut-il obtenir un rendez-vous **non-urgent** avec vous? (Veuillez sélectionner une réponse ci-dessous)

- Le jour même
- 2 à 7 jours (dans la semaine)
- 8 à 30 jours (dans le mois)
- Au delà de 30 jours
- Sans réponse

1. En général, de combien de semaines de **vacances par année** disposez-vous ? (Seuls des nombres peuvent être entrés dans ce champ)

semaines par année

1. Faites-vous partie d’une association/fédération professionnelle ? (Veuillez sélectionner une réponse ci-dessous)

- Oui
- Non
- Sans réponse

🖂 Hors ASCA et RME

Si non, pourquoi ?

# Environnement de travail

1. Quel est votre statut professionnel ? (*Veuillez sélectionner une réponse ci-dessous*)

- Thérapeute indépendant
- Thérapeute employé
- Les deux
- Sans réponse

1. Dans combien de cabinets exercez-vous ? (Cochez la ou les réponses)

- Aucun
- 1
- 2
- Plus que 2

1. Quelle situation correspond le mieux à votre environnement de travail ? (Cochez la ou les réponses)

- Je travaille seul dans un cabinet
- Je travaille dans un cabinet de groupe où nous partageons uniquement nos locaux
- Je travaille dans un cabinet de groupe où il existe une collaboration entre les différents thérapeutes présents au sein du cabinet
- Je travaille dans une permanence ostéopathique
- Autre

🖂 Si vous exercez dans plusieurs cabinets, merci de répondre pour chacun de ces cabinets

1. Partagez-vous votre cabinet avec d’autres thérapeutes de **médecine complémentaire** (ostéopathes/naturopathes/acupuncteurs y compris) ? (Veuillez sélectionner une réponse ci-dessous)

- Oui
- Non
- Sans réponse

Si oui, quelle est la profession des thérapeutes de médecine complémentaire avec lesquels vous partagez votre cabinet ? (Cochez la ou les réponses)

- Ostéopathe
- Ostéopathe assistant
- Acupuncteur
- Naturopathe
- Masseur
- Autre :

1. Partagez-vous votre cabinet avec des thérapeutes de **médecine conventionnelle** ? (Veuillez sélectionner une réponse ci-dessous)

- Oui
- Non
- Sans réponse

Si oui, quelle est la profession des thérapeutes de médecine conventionnelle avec lesquels vous partagez votre cabinet ? (Cochez la ou les réponses)

- Médecin
- Physiothérapeute
- Dentiste
- Sage-femme
- Autre :

# Patientèle

1. En moyenne, combien de consultations effectuez-vous **par mois** ?* (Seuls des nombres peuvent être entrés dans ce champ)

🖂SI vous ne souhaitez pas répondre, merci de noter 333.

1. En moyenne, combien de **nouveaux patients** traitez-vous **par mois** ? (Seuls des nombres peuvent être entrés dans ce champ)

🖂 Si vous ne savez pas, merci de noter 333

1. En moyenne, combien de **minutes** dure une de vos consultations ? (Seuls les nombres sont acceptés)

| Pour un nouveau patient |  |
| --- | --- |
|  |  |
| Pour un ancien patient avec un nouveau motif de consultation |  |
|  |  |
| Pour un suivi de traitement |  |

1. Pour un **même patient**, combien de consultations jugez-vous nécessaires en moyenne pour les motifs de consultations suivants ? (Seuls les nombres sont acceptés)

| Lombalgie aiguë (un épisode) |  |
| --- | --- |
| Cervicalgie aiguë (un épisode) |  |
| Lombalgie chronique (nombre de rendez-vous par année) |  |
| Cervicalgie chronique (nombre de rendez-vous par année) |  |

1. Selon votre estimation, quel est le **pourcentage de femmes** dans votre patientèle ? (Seuls des nombres peuvent être entrés dans ce champ)

pourcents

1. Globalement, quel **pourcentage** **de vos patients** fait partie des tranches d’âge suivantes? (Seuls les nombres sont acceptés)

| Enfants de 0-2 ans |  |
| --- | --- |
| Enfants de 3-18 ans |  |
| Adultes de 19-64 ans |  |
| Adultes de 65 ans et plus |  |

🖂 Merci de donner une estimation en pourcent. Il faudrait idéalement arriver à un total de 100%.

1. Avez-vous spécialisé votre pratique dans un des domaines ci-dessous ? (Cochez la ou les réponses)

- Non, je n’ai pas spécialisé ma pratique dans un domaine particulier
- Ostéopathie pédiatrique et/ou obstétrique
- Ostéopathie gynécologique et/ou périnéale
- Ostéopathie du sport
- Ostéopathie gériatrique
- Ostéopathie viscérale
- Ostéopathie crânienne
- Ostéopathie biodynamique
- Ostéopathie crânio-sacrée
- Autre: ______________________________________________

1. A quelle fréquence utilisez-vous les techniques suivantes ?*

|  | Jamais | Rarement | Parfois | Souvent | Très souvent | Je ne souhaite pas répondre |
| --- | --- | --- | --- | --- | --- | --- |
| Techniques structurelles (HVLA) appliquée uniquement au levier cervical |  |  |  |  |  |  |
| Techniques structurelles (HVLA) (sauf levier cervical) |  |  |  |  |  |  |
| Techniques fonctionnelles |  |  |  |  |  |  |
| Techniques musculaires |  |  |  |  |  |  |
| Techniques viscérales |  |  |  |  |  |  |
| Techniques crâniennes |  |  |  |  |  |  |
| Techniques tissulaires |  |  |  |  |  |  |
| Techniques fasciales |  |  |  |  |  |  |
| Techniques biodynamiques |  |  |  |  |  |  |
| Techniques crânio-sacrées |  |  |  |  |  |  |
| Techniques réflexes |  |  |  |  |  |  |

1. Pratiquez-vous d’autres techniques ? (Veuillez sélectionner une réponse ci-dessous)

- Oui
- Non

Si oui, lesquelles ?

1. A votre avis, quel pourcentage de vos patients ont une **assurance complémentaire** remboursant vos soins? (Seuls des nombres peuvent être entrés dans ce champ)

%

1. A votre avis, quel pourcentage de vos patients vous consulte pour une problématique relevant de la Loi fédérale sur l’Assurance Accidents (LAA) ? (Seuls des nombres peuvent être entrés dans ce champ) %

# Principaux motifs de consultations

1. Quelles sont les cinq raisons les plus souvent invoquées par vos patients pour venir consulter (les cinq motifs de consultation les plus fréquents) ?

| Motif principal N°1 |  |
| --- | --- |
| Motif principal N°2 |  |
| Motif principal N°3 |  |
| Motif principal N°4 |  |
| Motif principal N°5 |  |

🖂 *Merci de les lister en indiquant le motif de consultation le plus fréquent en premier*

1. En moyenne, dans le  **dernier mois**, à quelle fréquence vos patients sont-ils venus consulter pour les **motifs** **principaux** suivants ?

| Motifs de consultation | Jamais | Rarement | Parfois | Souvent | Très souvent |
| --- | --- | --- | --- | --- | --- |
| Check up ostéopathique (prévention ou contrôle) |  |  |  |  |  |
| Douleurs aiguës (0-4 semaines) |  |  |  |  |  |
| Douleurs subaiguës (4-12 semaines) |  |  |  |  |  |
| Douleurs chroniques (plus de 12 semaines) |  |  |  |  |  |
| Diminution de mobilité (sans douleur) |  |  |  |  |  |

1. A votre avis, quel pourcentage de vos patients souffrant de lombalgies aiguës vous consultent exclusivement? (Sans intervention d’un autre professionnel de la santé) ? (Seuls des nombres peuvent être entrés dans ce champ)

%

1. En général, durant l’année dernière, à quelle fréquence vos patients **adultes** sont-ils venus consulter avec les **motifs principaux** suivants  :

|  | Jamais | Rarement | Parfois | Souvent | Très souvent | Je ne souhaite pas répondre |
| --- | --- | --- | --- | --- | --- | --- |
| Céphalées, migraines |  |  |  |  |  |  |
| Cervicalgies |  |  |  |  |  |  |
| Dorsalgies, douleurs thoraciques, costalgies |  |  |  |  |  |  |
| Lombalgies, sciatalgies, cruralgies |  |  |  |  |  |  |
| Douleurs pelviennes (pubalgies, sacralgies, etc) |  |  |  |  |  |  |
| Douleurs dans les membres |  |  |  |  |  |  |
| Troubles digestifs |  |  |  |  |  |  |
| Vertiges |  |  |  |  |  |  |
| Trouble de la sphère ORL |  |  |  |  |  |  |
| Troubles orthodontiques (bruxisme, malocclusion, etc.) |  |  |  |  |  |  |
| Suivi de grossesse |  |  |  |  |  |  |

1. En général, durant l’année dernière, à quelle fréquence vos patients pédiatriques (0-18 ans) sont-ils venus consulter avec les motifs principaux suivants  :

|  | Jamais | Rarement | Parfois | Souvent | Très souvent | Je ne souhaite pas répondre |
| --- | --- | --- | --- | --- | --- | --- |
| Plagiocéphalie |  |  |  |  |  |  |
| Céphalées, migraines |  |  |  |  |  |  |
| Cervicalgies |  |  |  |  |  |  |
| Dorsalgies, douleurs thoraciques, costalgies |  |  |  |  |  |  |
| Lombalgies, sciatalgies, cruralgies |  |  |  |  |  |  |
| Douleurs pelviennes (pubalgies, sacralgies, etc) |  |  |  |  |  |  |
| Douleurs dans les membres |  |  |  |  |  |  |
| Troubles de la posture (scoliose, etc.) |  |  |  |  |  |  |
| Troubles digestifs (RGO, coliques, constipation, etc.) |  |  |  |  |  |  |
| Troubles de la sphère ORL (otite, angine, trouble de la succion, déglutition, etc.) |  |  |  |  |  |  |
| Troubles orthodontiques (bruxisme, malocclusion, etc) |  |  |  |  |  |  |

# Education des patients

1. Quel(s) thème(s) de **prévention** abordez-vous habituellement en consultation**?** (Cochez la ou les réponses)

- Aucun
- Hygiène posturale
- Tabac
- Problème d’alcool
- Mélanome
- Dépression
- Cancer du sein
- Obésité
- Autre

🖂 Si « Autre », merci de préciser

# Recherche scientifique

1. Etes-vous favorable à la recherche en ostéopathie ? (Veuillez sélectionner une réponse ci-dessous)

- Oui
- Non

1. Selon vous, quelle est l’importance des thèmes de recherche à aborder en ostéopathie ci-dessous ?

1=Extrêmement important 5=Pas du tout important

| Définition du profil des patients | 1 | 2 | 3 | 4 | 5 |
| --- | --- | --- | --- | --- | --- |
| Définition du profil des thérapeutes | 1 | 2 | 3 | 4 | 5 |
| Définition du rôle de l’ostéopathie au sein du système de santé | 1 | 2 | 3 | 4 | 5 |
| Etudes d’efficacité des traitements | 1 | 2 | 3 | 4 | 5 |
| Etudes des risques des traitements | 1 | 2 | 3 | 4 | 5 |
| Etudes des effets secondaires des traitements | 1 | 2 | 3 | 4 | 5 |
| Etude sur les mécanismes d’action des traitements | 1 | 2 | 3 | 4 | 5 |
| Etudes coût/ efficacité des traitements | 1 | 2 | 3 | 4 | 5 |

1. Souhaitez-vous contribuer à la recherche en ostéopathie ? (Veuillez sélectionner une réponse ci-dessous)

- Oui
- Non

1. De quelle manière souhaiteriez-vous contribuer à la recherche en ostéopathie ? (Cochez la ou les réponses)

- En participant à des recherches menées au cabinet avec des patients
- En répondant à des questionnaires
- En fournissant des données anonymes sur la patientèle
- Je ne souhaite pas répondre

# Place dans le système de santé

1. Pensez-vous que l’ostéopathie devrait être prise en charge par l’assurance obligatoire des soins (LAMal) ? (Veuillez sélectionner une réponse ci-dessous)

- Non
- Plutôt non
- Plutôt oui
- Oui
- Je ne sais pas

1. Pensez vous qu’une consultation d’ostéopathie devrait être proposée en milieu hospitalier ? (Veuillez sélectionner une réponse ci-dessous)

- Oui
- Non

Si oui, selon vous, dans quel secteur hospitalier l’ostéopathie devrait-elle être disponible en priorité ?

| **Propositions** |  | **Votre classement** |
| --- | --- | --- |
| Urgence |  |  |
| Rhumatologie |  |  |
| Oncologie |  |  |
| Médecine interne |  |  |
| Psychiatrie |  |  |
| Neurologie |  |  |
| Gynécologie/ obstétrique |  |  |
| Pédiatrie |  |  |
| Dermatologie |  |  |
| Traumatologie/ Orthopédie |  |  |
| Chirurgie |  |  |

Merci de classer les secteurs ci-dessus par ordre priorité, en commençant par le plus prioritaire. Merci d’en placer au moins cinq.
